# What is the impact of a research publication?

**DOI:** 10.1136/eb-2017-102668

**Published:** 2017-04-06

**Authors:** Seena Fazel, Achim Wolf

**Affiliations:** Department of Psychiatry, University of Oxford, Oxford, UK

**Keywords:** STATISTICS & RESEARCH METHODS, MENTAL HEALTH, PSYCHIATRY, MEDICAL EDUCATION & TRAINING

## Abstract

An increasing number of metrics are used to measure the impact of research papers. Despite being the most commonly used, the 2-year impact factor is limited by a lack of generalisability and comparability, in part due to substantial variation within and between fields. Similar limitations apply to metrics such as citations per paper. New approaches compare a paper's citation count to others in the research area, while others measure social and traditional media impact. However, none of these measures take into account an individual author's contribution to the paper or the number of authors, which we argue are key limitations. The UK's 2014 Research Exercise Framework included a detailed bibliometric analysis comparing 15 selected metrics to a ‘gold standard’ evaluation of almost 150 000 papers by expert panels. We outline the main correlations between the most highly regarded papers by the expert panel in the Psychiatry, Clinical Psychology and Neurology unit and these metrics, most of which were weak to moderate. The strongest correlation was with the SCImago Journal Rank, a variant of the journal impact factor, while the amount of Twitter activity showed no correlation. We suggest that an aggregate measure combining journal metrics, field-standardised citation data and alternative metrics, including weighting or colour-coding of individual papers to account for author contribution, could provide more clarity.

A number of developments in the metrics field have occurred in recent years, and, in this perspective article, we discuss whether they can inform how judgements are made about the impact of research papers in psychiatry and beyond.

The best-known approach has been to rely on journal impact factors, the most common of which is a 2-year impact factor, which calculates the average number of citations from articles published in the past 2 years of a particular journal.^1^ Many arguments against journal impact factors have been outlined, including the skewed nature of citations in most journals, the variation between and within fields (with basic science attracting more citations) and research designs (with systematic reviews being relatively highly cited) and the citation lag time in some research fields being longer than 2 years.^2^ A widely used alternative is the number of citations per paper, which can be drawn from research tools such as Scopus (http://www.scopus.com) and Google Scholar (scholar.google.com), with the latter including a broader range of citable items such as online reports and theses. The problem with citation counts is that they vary considerably by research area, and there have been recent attempts to account for this. One of these is the new iCite tool (icite.od.nih.gov) that normalises the number of citations of a particular paper to the median annual number of citations that NIH-funded papers in the field have received.^3^ Finally, alternative metrics have been increasingly used and include tools such as Altmetric (http://www.altmetric.com), which aims to capture the media and social media interest in a publication,^4^ and provides an overall article score and rankings compared with others in the same journal and/or time period.

A key problem with these approaches is that they do not account for an individual author's contribution to a paper, and therefore, high citation rates, h-indexes (for individuals) and iCite scores can be achieved for researchers who have not made significant contributions to a research area. The best example of this is being included as a coauthor of a large treatment trial or genetic consortium, where a researcher's contribution may be mostly in relation to participant recruitment. The Psychiatric Genomics Consortium workgroup for schizophrenia now average over 280 authors, although the number of authors varies considerably and is occasionally placed in the appendix. This highlights another problem with relying on measures of citation in that they do not account for the total number of authors. Accordingly, h-indexes should be routinely provided for papers where the author is first, corresponding or last author (and second author in psychology).

Another limitation is that some of these metrics are subject to measurement error, and some can be gamed. To take an example of the latter, Altmetric scores can potentially be artificially increased by robots that repost press releases. At the same time, they may not pick up all media activity if the article is not cited accurately or embedded in a hyperlink. In addition, they are considerably higher in studies on exercise, diet and lifestyle, and in areas that attract controversy.^5^ Although some research has shown some correlation between alternative metrics and citations scores,^6^ particularly early on after publication, they do not account for the inherent problems outlined above about the extent of an individual's contribution, normalisation by field and measurement error.

An important natural experiment has been undertaken in the UK where a very large sample of papers (k=148 755) was investigated against a gold standard of peer review as part of the 2014 Research Exercise Framework (or REF 2014). The REF was a national exercise undertaken to assess research from 2008 to 2013 in higher education institutions in the UK, which succeeded an earlier process (called the Research Assessment Exercise in 2008). It determined the extent of central government basic research funding for these institutions until the time of the next evaluation (thought to be in 2021). Three factors were considered—outputs (which made up 65% of the overall quality profile), impact (20%) and environment (15%). A detailed bibliometric analysis of the output data was published and provides a breakdown by unit of assessment.^7^ Each eligible academic typically submitted four outputs for the REF. Here, we will discuss the assessment block that most departments of psychiatry and psychology will have entered, namely Unit 4 (Psychiatry, Clinical Psychology and Neurology), which assessed 9086 journal articles. The analysis took 15 different metrics for each paper and analysed to what extent, they were correlated with the final view of the REF panel (which was made up of an expert committee of 39 researchers). The strongest bivariate correlations between these metrics and scoring the highest score per paper are presented in figure 1. Each paper was measured against a standard of originality, significance and rigour and the best papers were scored a 4*, which represented ‘world leading’, whereas a 3* reflected ‘internationally excellent in terms of originality, significance and rigour but which falls short of the highest standards of excellence’. Lower scores of 2*, 1* and unclassified were also given.

**Figure 1:**
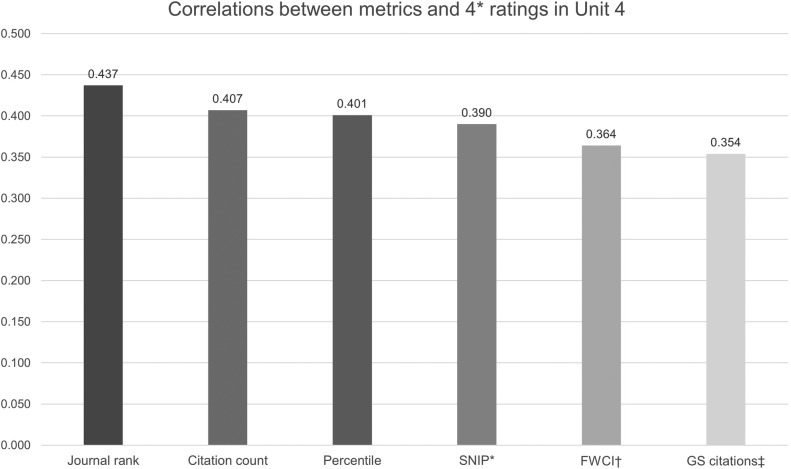
Strongest bivariate correlations between paper metrics and the highest REF score. *Source-Normalised Impact per Paper; †field-weighted citation impact; ‡Google Scholar citations.

The strongest correlation was with the SCImago Journal Rank, which is a metric based on the notion that not all citations have equivalent weight, and categorises journals per field into four categories (from low to high rank). It assumes that the subject field, quality and reputation of the journal have a direct effect on the value of a citation. This was followed by the absolute number of Scopus citations and the percentile of highly cited publications. The Source-Normalised Impact per Paper attempts to relativise the citation impact by weighting citations based on the total number of citations in a subject field. Thus, the impact of a single citation is given more value in participants where citations are less likely.

Notably, there was no correlation between Twitter activity generated by an article and a top REF score (of ‘4*’), and associations were weak for full-article requests, downloads and reads on one platform (Mendeley, http://www.mendeley.com). Interestingly, for the overall REF which included 36 units, the 3 strongest markers of quality were the SCImago Journal Rank, the Source-Normalised Impact per Paper and the percentile. Correlations tended to be stronger in the sciences than in the arts and humanities.

What this suggests is that the judgement of an expert panel that was constituted to examine a paper's impact, perhaps the closest to a gold standard that is possible, was most strongly correlated with the SCImago Journal Rank, which itself is based mostly on the journal impact factor. This is not surprising as many such journals have more stringent peer and statistical review, insist on adhering to research guidelines, benefit from professional editors, and articles in high-impact journals are often cited to add legitimacy to a particular field of study, and may be included in introductions. Further, the analysis of the REF 2014 suggests that new metrics appear unlikely to replace simpler ones such as journal impact factor and number of citations per year.

So where does this leave someone trying to assess the impact of a paper? As there are difficulties with relying on one metric, we suggest that a combination of metrics should be used. We recommend that those most correlated with expert judgement take priority but can see a role for Altmetrics, with the caveats noted above, as a measure of wider public engagement, impact and interest. In the future, a combined score that takes into account journal impact factor, number of citations, iCite, Altmetric scores and a different colour coding or weighting for those papers where authors have made a substantial contribution (eg, where an author has been first/last/corresponding) would assist in providing some clarity.
